# Composition-Tunable Properties of Cu(Ag) Alloy for Hybrid Bonding Applications

**DOI:** 10.3390/ma16237481

**Published:** 2023-12-02

**Authors:** Sarabjot Singh, Kathleen Dunn

**Affiliations:** Department of Nanoscale Science and Engineering, University at Albany, State University of New York, Albany, NY 12203, USA; ssingh24@albany.edu

**Keywords:** pulsed electrochemical deposition (PED), Cu(Ag) alloy, thin films, hybrid bonding interconnections

## Abstract

In the present study, the properties of Cu(Ag) alloy films were studied to evaluate their potential use as an alternate material for interconnection in hybrid bonding. Thin alloy films of Cu(Ag) were deposited by pulsed electrochemical deposition (PED) using a sulfuric acid-based bath, rotating disk electrode, and hot entry. Secondary ion mass spectrometry (SIMS) was used to measure the silver content of the films, with us finding that it decreases with increasing duty cycle. Thereafter, bright field scanning transmission electron microscope (STEM) imaging in combination with energy-dispersive x-ray spectroscopy (EDS) was used to visualize the thin film microstructure and to confirm the uniform distribution of silver throughout the film, with no bands being seen despite the pulsed nature of the deposition. Film resistance was measured by a four-point probe to quantify the impact of Ag content on resistivity, with us finding the expected linear relationship with the Ag content in the film. Furthermore, the coefficient of thermal expansion (CTE) of the films was measured using X-ray diffraction, and modulus and hardness were measured via nanoindentation, revealing linear dependences on the Ag content as well. Notably, the addition of 1.25 atom% Ag resulted in a significant increase in the CTE from 17.9 to 19.3 ppm/K, Young’s modulus from 111 to 161 GPa, and film hardness from 1.70 to 3.99 GPa. These simple relationships offer a range of properties tunable via the duty cycle of the pulsed plating, making Cu(Ag) a promising candidate for engineering wafer-to-wafer metal interconnections.

## 1. Introduction

Three-dimensional integration (3DI) technology has garnered significant attention for achieving high speed, high density, low power consumption, improving circuit performance, and keeping Moore’s law alive [1,2,3]. The major advantage of 3DI is the opportunity for heterogeneous integration, that is, integrating different types of semiconductor devices such as sensors, processors, and memory on a single platform [4]. 3DI is typically achieved by stacking multiple chips vertically together into a single package, with the components inside that package connected using hybrid bonding or through-silicon vias (TSVs). In wafer-to-wafer (W2W) hybrid bonding technology, both the dielectric and embedded metal are bonded permanently to form the interconnection, offering a comparatively cost-efficient solution for high-performance integration without the need for expensive through-silicon vias [5,6]. In the past few decades, many studies have been conducted to continuously improve bonding technology performance. However, the industry is still facing numerous challenges to achieve robust and successful hybrid bonding [5,7].

**Figure 1:**
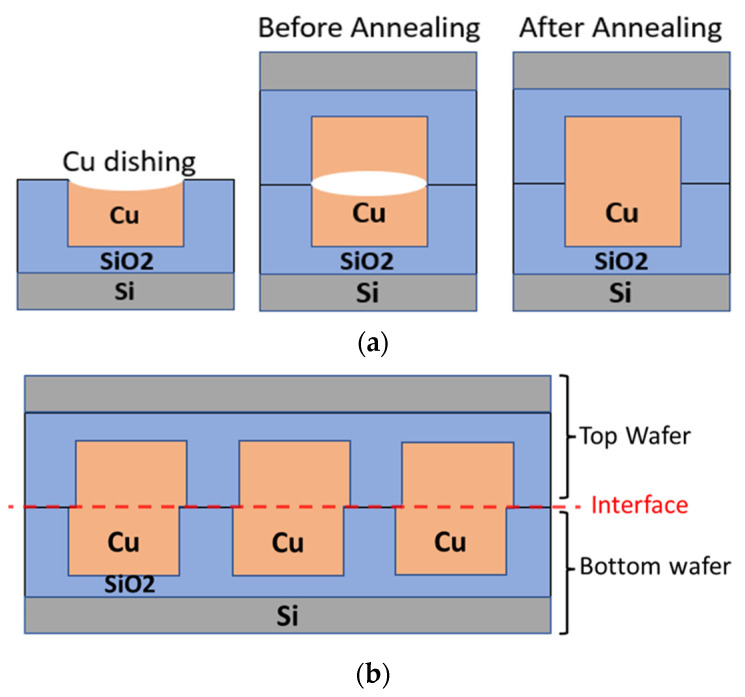
(**a**) Schematic drawing of hybrid bonding process flow. (**b**) Schematic drawing of misalignment at the bonding interface between the top wafer and bottom wafer.

One significant challenge in the quest for simultaneous bonding across the interface is the recessed surface of the metal, i.e., dishing, caused by chemical–mechanical planarization (CMP), as shown in Figure 1a. If this dishing is small, it may be overcome by a thermal anneal, as the CTE for copper is greater than that of the surrounding dielectric, allowing the copper to expand to fill the gap [8]. However, temperatures in excess of 350 °C would be needed to cause enough expansion to overcome typical dishing values. These temperatures can be problematic for front-end devices and also allow for slippage and bond alignment issues during hybrid bonding. For example, it has previously been reported that plastic deformation can cause misalignment (Figure 1b) and weakness at the bond interface [7].

Ideally, a solution might be found for both of these issues by using a material with a higher CTE and higher resistance to plastic deformation (i.e., higher yield strength than pure copper). This may be accomplished by decreasing Cu grain size, for example, or alloying Cu with other suitable metals at the expense of higher electrical resistance.

Cu alloys have been considered of great interest due to their improved properties and versatile applications in fields like additive manufacturing, batteries, and electrical wiring [9,10,11]. Various materials alloyed with Cu such as cobalt (Co), manganese (Mn), silver (Ag), and tin (Sn) have been explored as a result of some of their unique advantages over pure Cu, including higher activation energy for Cu self-diffusion, superior resistance to corrosion, and improved electromigration lifetimes [12,13,14,15,16,17]. However, little work has explored the application of such alloys in hybrid bonding interconnection. Keeping the requirements of this specific application in mind, we explore the use of Ag solute to increase the CTE and reduce the plasticity in the Cu matrix. Cu(Ag) has previously been studied for wiring applications because its impact on resistance is minimal compared to the other alloying metals [15,18], and it has also been reported to have a slight tendency for improved resistance to electromigration [17]. Here, we report the microstructure, electrical, thermal, and mechanical properties of Cu(Ag) alloys as an alternative to pure Cu for hybrid bonding. The resulting insights are discussed in terms of better engineering the alloy material properties in anticipation of its integration into semiconductor manufacturing.

## 2. Experimental Section

### 2.1. Film Growth

**Figure 2:**
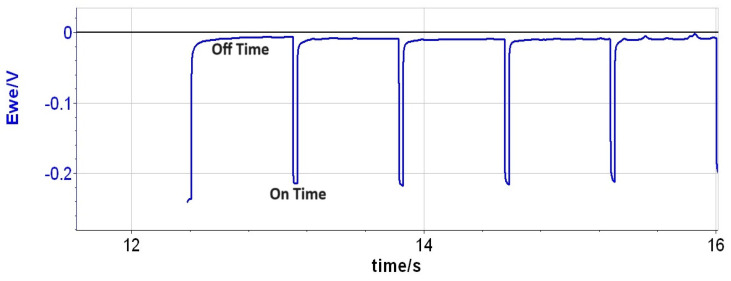
Example of the pulse plating profile used for the deposition of Cu(Ag) alloys.

Thin alloy films of Cu(Ag) were co-deposited by pulsed electrochemical deposition (PED) in a sulfate electrolyte adapted from Volov et al. [19]. Silver ions have limited solubility in this bath because the chloride ions necessary for copper plating combine with Ag ions to form AgCl precipitates. To overcome this limitation, PED applies a pulsed voltage waveform to the working electrode during the deposition process as shown in Figure 2. During the ON portion of the pulse, the current is held at 20 mA/cm^2^. During the OFF time, the voltage approaches 0 V, allowing Ag ions to displace some Cu atoms, creating a surface “monolayer” of Ag atoms via an exchange reaction. Subsequent deposition of copper during the next ON portion of the cycle covers the Ag, creating the alloy. The ratio of Ag to Cu in the alloy films can be regulated by adjusting the duty cycle (DC) of the pulse, that is, the ratio of the ON time of the electroplating to the total cycle time. Changing the DC modulates the ratio between the ON and OFF times, with a higher DC creating thicker Cu layers before the Ag ions are allowed to exchange with the Cu surface atoms, resulting in a lower overall concentration of Ag in the alloy.

**Figure 3:**
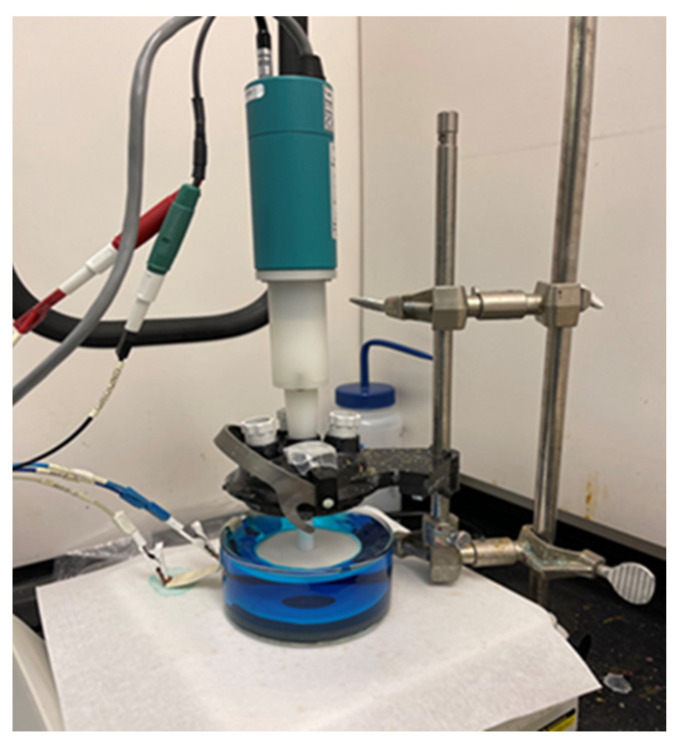
Reaction setup used for electrodeposition.

Blanket substrates were procured from an industry-standard 300 mm wafer processing line at NYCREATES and comprised a (100) silicon wafer with native oxide, followed by an 8 nm I-PVD TaN/Ta liner and then ~20 nm I-PVD Cu(Mn) as the seed layer necessary for subsequent electrochemical depositions. The wafers were cleaved into approximately 2.5 cm × 2.5 cm coupons which were then mounted on a rotating disk electrode (RDE) (as shown in Figure 3). The coupon was prepared by cleaning the surface and cutting a circular hole in the insulating tape, leaving a surface area of 1.98 cm^2^ of the copper seed layer exposed for the deposition process. The substrate attached to the RDE was held at a potential prior to and during entry to prevent the dissolution of the seed layer in the acid bath, known as hot entry. Once the coupon was immersed in the electrolyte solution and the current was detected, the power supply switched to galvanostatic control, where a constant current of the desired amount was applied. The electrolyte solution used contained 0.3 M sulfuric acid, 0.64 M copper sulfate, 1.7 µM silver nitrate, and 10 ppm hydrochloric acid dissolved in 200 mL of DI water. The deposition process was controlled using electrochemical software called EC-Lab (version 11.02) and a potentiostat/galvanostat electrochemical workstation from BioLogic Instruments (Seyssinet-Pariset, France). Although the film thickness was not independently monitored, Faraday’s law was used to calculate the desired total charge to be deposited in the exposed area, which is related to thickness via the density of (bulk) copper films. After the deposition process was complete, the coupon was rinsed with deionized water and blown dry with nitrogen gas.

### 2.2. Film Characterization and Measurements

#### 2.2.1. Composition Analysis

The compositions of the Cu(Ag) thin films were characterized using secondary ion mass spectrometry (SIMS) in a Physical Electronics (PHI) 6650 quadrupole SIMS instrument by Physical Electronics, Inc. (Chanhassen, MN, USA). Depth profiles were collected using primary ion bombardment with 5 keV Cs+ at an incidence angle of 60° from normal and the detection of secondary ions with negative polarity. Dynamic SIMS spectra were acquired to evaluate carbon and oxygen impurity levels and silver content. Raw ion counts were converted to atomic concentrations by using a Cu reference standard with known ion-implanted quantities of Ag. The calculated concentrations should be accurate to within the estimated error of 15%. The primary ion beam was rastered over 700 µm^2^, and the sputtered area formed a crater whose depth was measured using a Veeco Dektak 150 stylus profilometer manufactured by Veeco Instruments Inc. (Plainview, NY, USA) to determine the erosion rates during SIMS analysis, with an estimated error of 5%.

#### 2.2.2. Microstructure and Solute Distribution Analysis

Cross-sectional TEM lamellas were prepared in an Nova NanoLab 600 Dual Beam Focused Ion Beam-Scanning Electron Microscope (FIB-SEM) by FEI (Hillsboro, OR, USA). The SEM column was operated at 5 kV for imaging while the FIB column was operated at 30 kV for cross-sectioning. Bright-field scanning transmission electron microscopy (BF-STEM) using a Titan3 80-300 (S)TEM (from FEI, Hillsboro, OR, USA) operated at 300 keV was employed to determine the microstructure of the Cu(Ag) alloy films. The solute distribution in the alloy films was determined using the elemental mapping function of a SuperX energy-dispersive X-ray spectroscopy (EDS) system by Bruker (Billerica, MA, USA) in the STEM, with a probe current of 0.5 nA.

#### 2.2.3. Electrical Measurements

The sheet resistance of the Cu(Ag) alloy films was measured using a QuadPro Four probe measurement system (model: QPS-A8), manufactured by Lucas/Signatone Corporation (Gilroy, CA, USA).

#### 2.2.4. Mechanical Testing

Prior to mechanical testing, the films were planarized by chemical mechanical polishing (CMP) using silica slurry with a particle size of 0.05µm to minimize the influence of surface roughness on the measured mechanical properties. The resulting surface roughness was measured using atomic force microscopy (AFM) manufactured by Bruker (Billerica, MA, USA).

After CMP, nanoindentation experiments were performed with a Hysitron TriboIndenter from Bruker (Billerica, MA, USA). with a load resolution of 100 nN, a displacement resolution of 1 nm, and a maximum indentation depth of 5 µm. For all experiments, a Berkovich tip (TI-0039, Hysitron) with a radius of 100 nm was used. Quasi-static nanoindentation measurements were performed on a fused quartz standard (S/N 5–0098, Hysitron) to calculate the contact area function parameters, as instructed by the manufacturer. Before the nanoindentation experiments, the standard fused quartz was also tested (E_τ_ = 69.9 ± 5% and H = 9.1 GPa ± 10%).

The indentation experiments were performed at room temperature on the samples with a displacement-controlled function (50 nm) with 5 s of loading, 2 s holding, and 5 s of unloading time. The reduced modulus (E_τ_) and hardness (H) were calculated using the Hysitron TriboIndenter’s data analysis software (Triboscan version 9.8.2.1) from the unloading segment. A total of 10 indents were performed for each sample to obtain reliable and representative data. The average values of hardness and modulus were determined from these multiple indentations. This approach ensures accurate and statistically significant results, allowing for a comprehensive understanding of the mechanical properties of the Ag-free Cu and Cu(Ag) alloy films.

#### 2.2.5. Coefficient of Thermal Expansion (CTE)

The CTE was calculated from the lattice parameter measured by X-ray diffraction during heating. The diffractometer is an Empyrean made by Malvern Panalytical (Malvern, UK) and uses Cu K_α_ X-rays. The high-temperature stage equipment used was an HTK 1200N. X-ray diffraction measurements were performed in the temperature range from 50–350 °C in 50 °C increments with a ramp rate of 5 °C/min followed by a 5 min hold at the specific temperature for the sample to reach equilibrium before the diffraction scan began. Each scan was from 25–100° 2θ with a 0.0066° step size and the scan speed was about 4.9° per minute with a PIXcel3D detector. Data were analyzed by performing Rietveld refinements with HighScore Plus.

## 3. Results and Discussion

### 3.1. Alloy Composition and Microstructure

**Figure 4:**
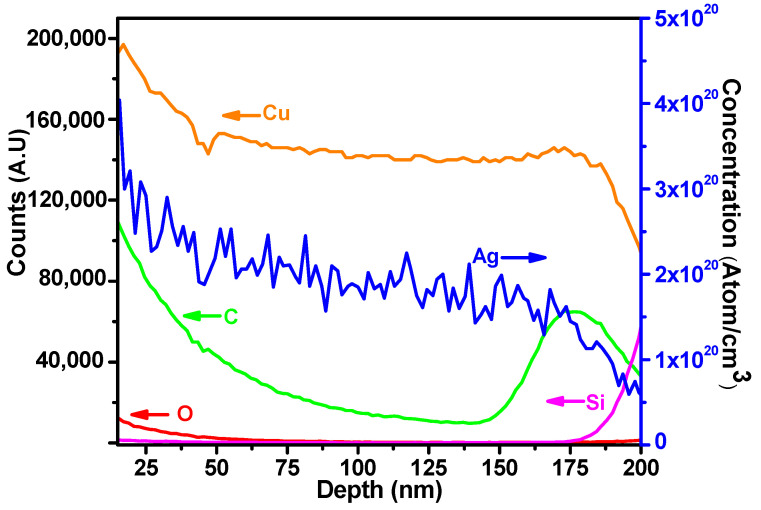
SIMS depth profile for a sample deposited with 4 DCs. The left axis shows the signal counts, whereas the right axis shows the Ag concentration determined using an implanted standard.

For the analysis of film deposition conditions and Ag content, secondary ion mass spectrometry (SIMS) was used. All of the samples were measured for alloy composition using the SIMS depth profile. An example is shown in Figure 4, for the case of a sample with four DCs (pulse ratio of 4%). In this depth profile, the surface of the film is at the far left, with the sputter depth plotted on the horizontal axis and the signal counts plotted on the left vertical axis. Near the surface, the composition of all elements is higher than in the bulk, suggesting some enhanced ion yield due to the presence of surface oxygen. After a depth of ~50 nm, the copper signal stabilizes, and the influence of surface oxygen is diminished. The Ag signal in this region was used to determine the silver content of the film via an implanted standard and the concentration on the vertical axis to the right, in blue. Despite the pulsed deposition, no periodic fluctuations in the silver content were noted.

**Figure 5:**
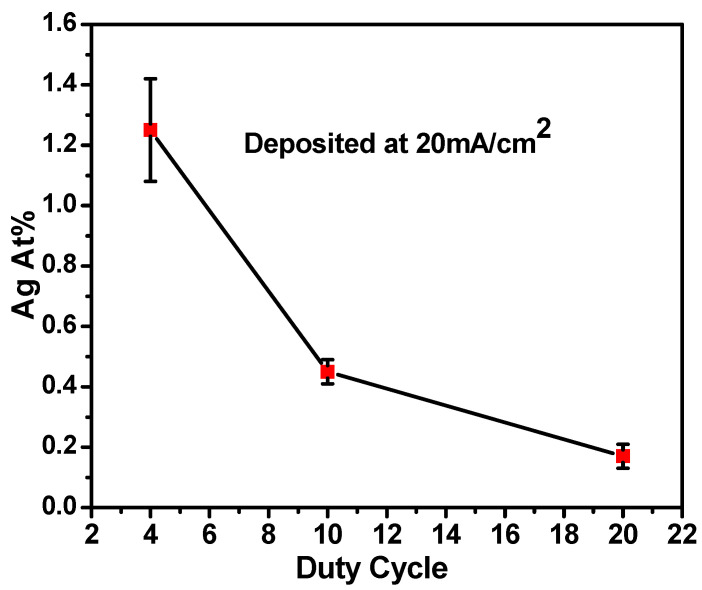
Ag content vs. duty cycle for films deposited with a current density of 20 mA/cm^2^. As anticipated, a lower duty cycle allows for a longer window for the displacement reaction, resulting in higher Ag incorporation.

The Ag content of a series of films was then plotted with respect to the duty cycle as shown in Figure 5, with it generally aligning with expectations. Table 1 shows the values of Ag content obtained from all four samples listed with respect to the deposition conditions. Specifically, films with lower duty exhibited higher Ag content because the increased length of the ‘off’ portion of the cycle allows more time for the displacement reaction to incorporate Ag into a film. These findings provide the ability to precisely control Ag content without modifying the fixed electrolyte composition containing Cl^−^ ions as Ag^+^ remains insoluble. Within the range studied, the 20 mA/cm^2^ with four DCs gave the highest Ag concentration. These conditions were subsequently employed to assess the microstructure and solute distribution in the Cu(Ag) alloy films.

**Figure 6:**
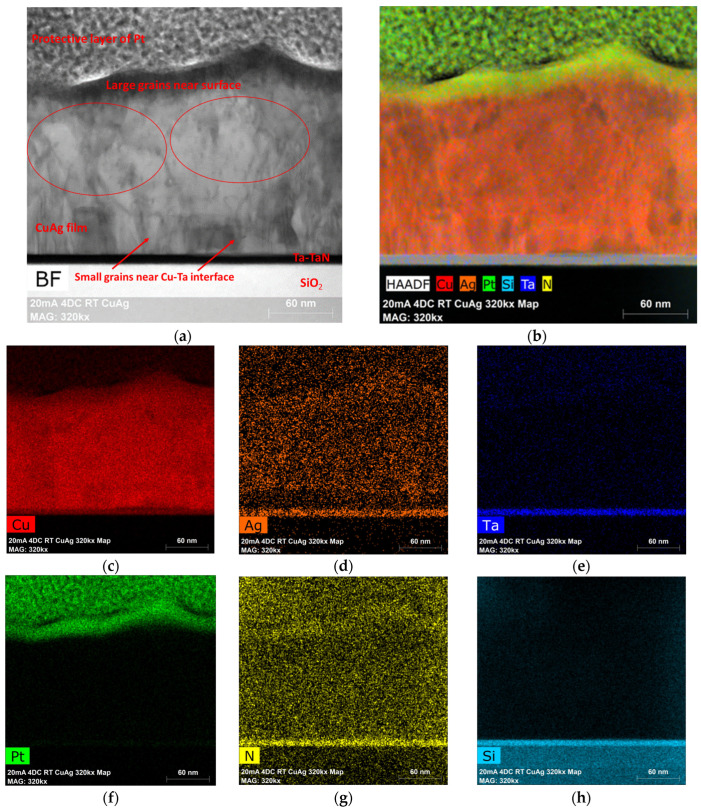
(**a**). BF-STEM image of the cross-section of the as-deposited Cu(Ag) alloy film deposited at 4 DCs. The microstructure suggests that the surface roughness is due to the large grains of the as-deposited film. (**b**). The EDS overlay on the HAADF-STEM image of Cu(Ag) alloy films confirms a uniform distribution of Ag throughout the film thickness without any obvious precipitates or segregation. (**c**–**h**) EDS maps of Cu, Ag, Ta, Pt, N, and Si showing the individual distribution of these elements.

Figure 6a shows the BF-STEM image of the cross-section of a typical Cu(Ag) film. This image shows the rough surface of the deposited films, with a thickness ranging from 160–200 nm, closely aligning with our targeted thickness of 200 nm. The microstructure near the Cu-Ta interface of the film exhibits numerous small grains consistent with the high nucleation density typical of electroplated copper films. Larger grains emerge (circled) as the film increases in thickness, with differing growth rates and surface terminations responsible for the surface roughness of the film. This rough film surface is different from the typical electrochemical deposition of Cu films which are deposited under high nucleation density conditions, yielding a smooth film. In the case of these PED films, the surface roughness implies that the large grains constitute the as-plated microstructure. These large as-plated grains are a consequence of the extended off-times associated with the PED process, allowing ample time for surface atom diffusion to occur, ultimately settling on the lowest energy addition sites on the nucleated grains [19,20].

**Figure 7:**
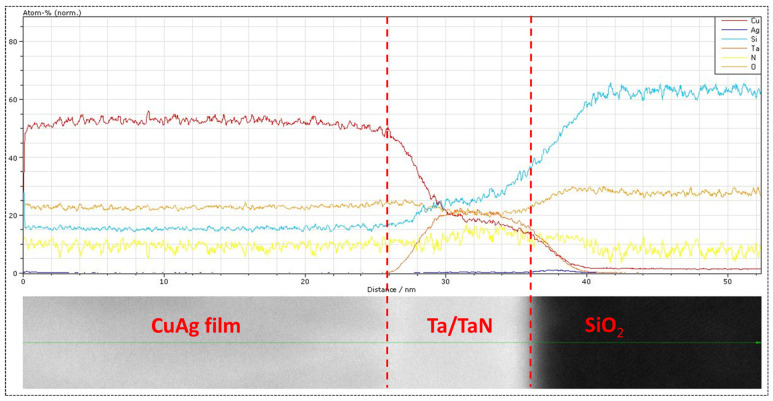
EDS line scan for Cu(Ag) alloy film deposited at 4 DCs showing a uniform concentration of Cu and Ag with no periodic banding or peaks.

To analyze the distribution of Ag solute in the alloy films, EDS elemental mapping was performed. Although the SIMS data indicate that the overall average composition of this film was 1.25%, the pulsed nature of the deposition process could result in bands of higher and lower silver content corresponding to the OFF and ON portions of the cycle. SIMS was unable to confirm or exclude this possibility due to the roughness of the film surface, which is replicated during the sputter depth profiling, meaning that a horizontal band (parallel to the substrate surface) is not entirely sampled simultaneously in SIMS. However, these higher concentration bands (if present) should be easily detectable through EDS. Figure 6b, an EDS overlay on the HAADF image with 7.5 million counts, shows a uniform distribution of Ag throughout the entire thickness of the sample, without any noticeable banding despite the pulsed plating process. Figure 6c–h breaks out each elemental map separately. In some cases, peak overlaps result in apparent interaction (see, for example, Figure 6d, where it appears there is Ag in the barrier layer). This is an unfortunate problem in EDS mapping due to the close proximity (in energy) of Ag, Ta, and N peaks and similarly for the Si and Ta peaks. This near overlap in the EDS data may give the impression of an interaction when none exists and is a known limitation of EDS. Away from these overlaps, the EDS analysis revealed the presence of O, C (not shown here), and N impurities dispersed throughout the sample thickness. In addition, a line scan, shown in Figure 7, was performed near the bottom of the sample. Both Cu and Ag were found to be present at the constant rate without any observable spike throughout the line scan until the Ta/TaN/Si interface was reached, confirming a uniform distribution with no periodic banding. Similarly, no small precipitates or grain boundary segregation was noted within the Cu(Ag) film, which is undetectable by SIMS because its analysis beam is larger than the grain size of these films.

### 3.2. Mechanical Properties

**Figure 8:**
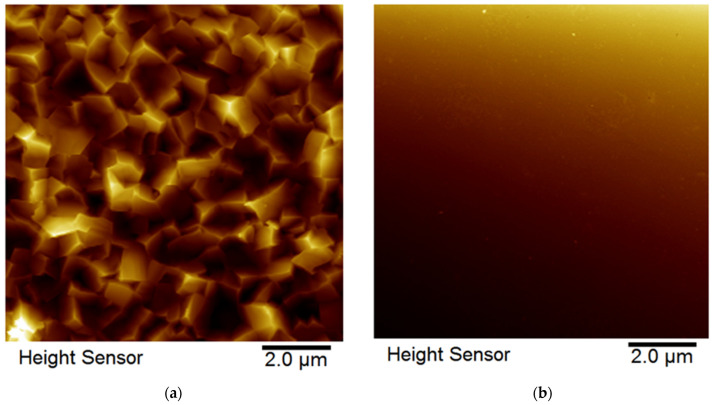
Surface roughness measured by AFM: (**a**) for an as-deposited sample using 4 DCs showing a significantly rough surface and (**b**) smooth surface obtained after CMP polishing.

The images shown in Figure 8a,b depict the surface morphology of the films before and after CMP treatment, specifically for the 20 mA/cm^2^ 4 DCs condition. The initial surface exhibited a high roughness of 130 nm, which was significantly reduced by polishing to a final roughness of 3 nm. This surface preparation step was necessary to ensure that any variations in mechanical properties can be attributed primarily to the alloy composition rather than surface roughness effects.

**Figure 9:**
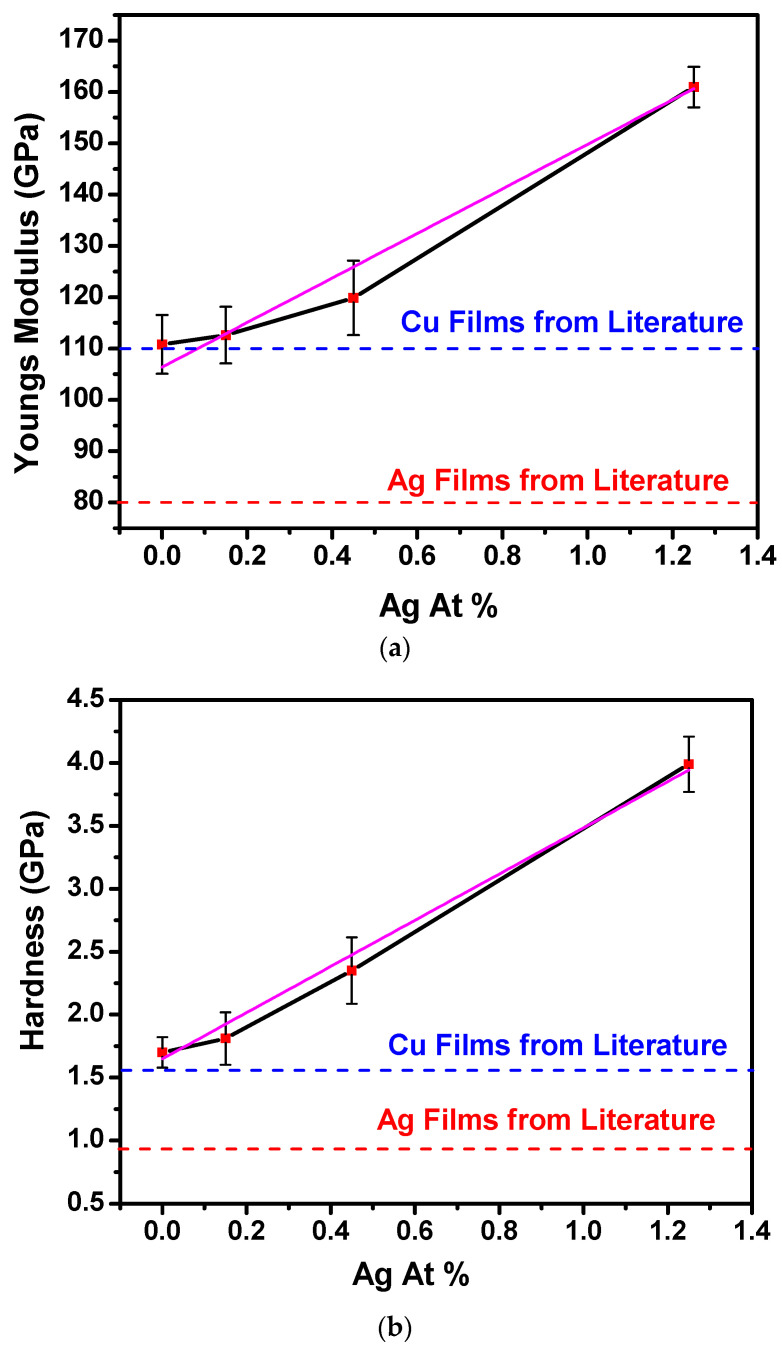
(**a**). Modulus vs. Ag content of the Cu(Ag) alloy films showing the modulus values linearly increasing with the increase in the Ag content of the films. (**b**). Hardness vs. Ag content of the Cu(Ag) alloy films showing the hardness values linearly increasing with the increase in the Ag content of the films. Black lines connect mean values, with linear fit plotted in purple.

After the CMP treatment, nanoindentation measurements were performed to measure the modulus and hardness values. Table 2 shows the values obtained from the four samples listed. Figure 9a,b plots these data as a function of silver content, along with values for pure copper films and pure silver films extracted from the literature reports [21,22]. Surprisingly, the Cu(Ag) films exhibited higher Young’s modulus and greater hardness than either the pure Ag or pure Cu films, with values that increased linearly with the Ag content.

Because the indentation depth was ~25% of the film thickness, additional testing was carried out to assess whether the measured hardness was being influenced by the proximity of the substrate. A 1 μm thick film of pure Cu was tested, and it revealed a hardness of 110 GPa, consistent with the values in Table 2 for the thinner films (within the margin of error for the test). These additional data provide a crucial reference point to rule out the substrate effect as the source of the observed hardness of these thin films.

These values were particularly surprising given the low solute content. A mere 1.25% Ag resulted in significantly higher modulus and hardness values of 161 GPa and 3.99 GPa, respectively. These remarkable enhancements of the mechanical properties are consistent with the solution and precipitation strengthening mechanism in the Cu(Ag) alloy [23]. However, no precipitates were observed in the STEM-EDS analysis. This indicates that the observed mechanical behavior may be attributed to solution hardening, where the dissolution of solute atoms within the Cu matrix contributes to the enhanced strength and hardness.

This effect arises because the atomic radii of Cu and Ag are 1.28 Å and 1.75 Å, respectively. The substitution of Ag on the Cu lattice causes strain which impedes the motion of dislocations, which manifests as an increase in measured hardness. This mechanism increases the yield strength (and hardness) with a square root dependence on the solute concentration, but for these dilute films, we suggest that the square root can be reasonably approximated with a linear trend. Given the small grain size of our films, the Hall–Petch effect could contribute to a higher hardness than bulk values as well, but we deem this to be a minimal contribution since the Ag-free film has a hardness comparable to other literature reports, and we see no evidence that the addition of Ag decreased the grain size further.

Regardless of the mechanism, the results are promising for targeted application in hybrid bonding because yield strength σ is related to hardness (H) in this regime (nanoindentation depths between 50–2000 nm) through the linear relationship:

H = c′ σ + b(1)

where c′ and b are parameters associated with indentation displacement [24].

Thus, higher hardness implies better resistance to plasticity, as desired for improving mechanical behavior.

### 3.3. Thermal Properties

**Figure 10:**
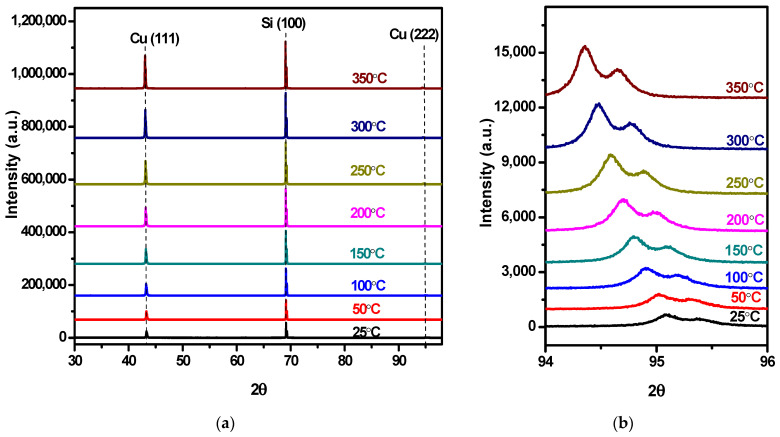
High-temperature XRD patterns of Cu(Ag) alloy films with 1.25% Ag obtained at different temperatures where: (**a**) the peak at 43.2° corresponds to Cu(111) and the one at 69.25° corresponds to Si(100) from the substrate; (**b**) zoomed-in view of the Cu (222) XRD peak at 95° showing a systematic shift in the Cu(222) peak towards lower angles with increasing temperature.

High-temperature X-ray diffraction measurements were conducted on Cu(Ag) alloy films with varying compositions to measure their lattice parameter as a function of temperature, with the stage height adjusted to compensate for the thermal expansion of the holder, at all temperatures. All refinements were carried out under the assumption that the sample height was adequately corrected by the data collection software (called Data collector by Malvern Panalytical, version 6.1b) and checked after refinements were complete. No change in the sample height in the sample displacement parameter was needed, suggesting that the assumption of adequate correction by the automated software was justified. Figure 10a illustrates the XRD patterns of Cu(Ag) alloy films containing 1.25% Ag. The XRD patterns show the reflections from three characteristic crystal planes: Cu(111) at 43.2°, Si(100) at 69.25°, and Cu(222) at 95.0° 2θ. This latter reflection, having low intensity, is expanded in Figure 10b, with the two peaks attributed to the Kα_1_ and Kα_2_ doublet of the X-ray source. The (222) peak shifts leftward with increasing temperature, corresponding to a larger interplanar spacing attributed to thermal expansion. As the temperature rises, the peaks become sharper and more intense, consistent with crystallite growth and the relief of microstrain during heating, resulting in more distinct diffraction peaks.

The lattice parameter was calculated at each temperature and used to calculate the thermal expansion coefficient of each film.

The linear thermal expansion coefficient, αL, along a length dimension ‘a’ is defined by Equation (2):

(2) $$\alpha_{L}=(\frac{1}{a_{0}})*(\frac{\Delta a}{\Delta T})$$

where $\alpha_{L}$ is the linear thermal expansion coefficient, a_0_ is the original lattice parameter, Δa is the change in lattice parameter, and ΔT is the change in temperature. This can be rearranged to:

(3) $$\frac{\Delta a}{a_{0}}=\alpha_{L}*\Delta T$$

**Figure 11:**
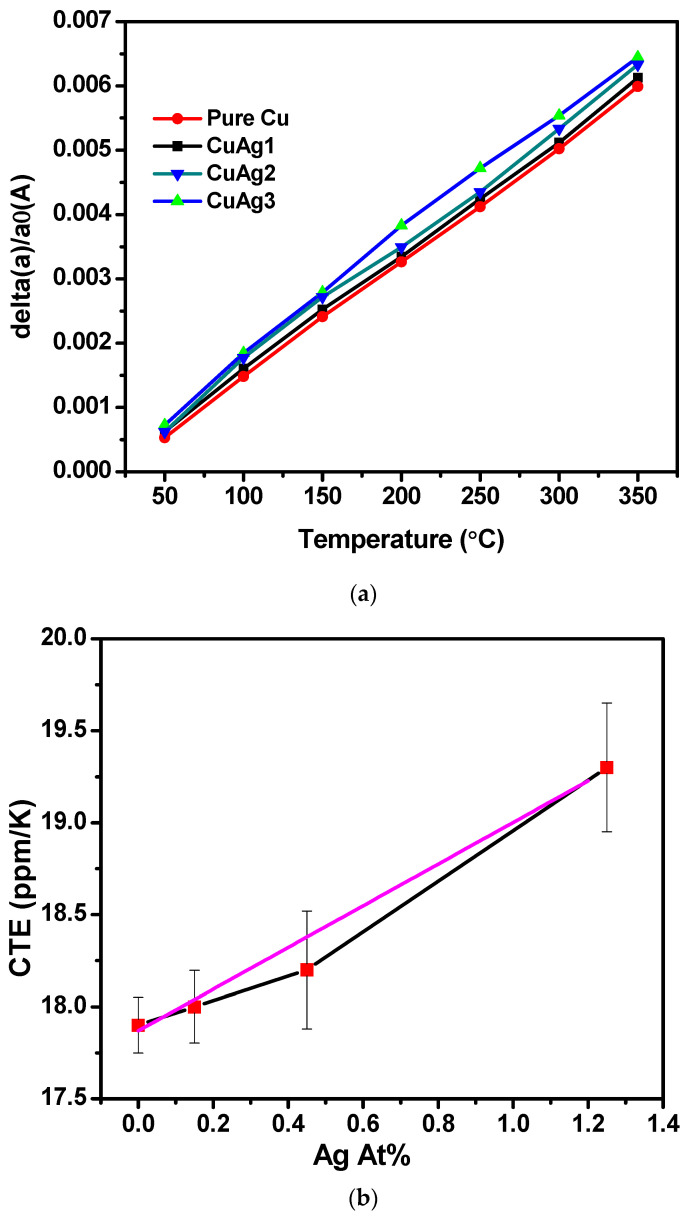
(**a**). Temperature dependence of the ratio of change in the lattice parameter compared to the original value. (**b**). The calculated CTE vs. Ag content depicts a linear dependence on composition. Black lines connect mean CTE values, with a linear fit plotted in purple.

Plotting the relative change in lattice parameter (Δa/a_0_) versus temperature allows the thermal expansion coefficient to be extracted as the slope of this relationship, as shown in Figure 11a. All samples showed the expected linear relationship, and the CTE value for the Ag-free Cu film (17.9 ppm/K) is consistent with the literature value of 17.7 ppm/K for copper films [25]. The CTE values of the alloys determined from the respective trendlines are higher than that of Ag-free copper and are tabulated in Table 3, showing a gradual change in the CTE with silver content. This can be seen more clearly in graphical format in Figure 11b.

The CTE shows a linear dependence on composition, with a mere 1.25% Ag resulting in an 8% increase in the CTE. This considerable impact is consistent with a previous study that reported pure Ag films on SiO_2_ substrates exhibiting a significantly higher CTE value of 31 ppm/K compared to the bulk CTE value of Ag at 19 ppm/K [26]

This can be attributed to the lateral constraint within the thin films due to the presence of the Si substrate, leading to anisotropic expansion behavior. The film’s restricted expansion in the x- and y-directions combined with its freedom to expand solely in the z-direction could be related to Poisson’s ratio, which characterizes how materials deform under stress. Using a Poisson ratio of 0.33 for copper, we can calculate the expected vertical expansion of the film if fully constrained by the silicon substrate and its lower CTE (2.6 ppm/K). This would predict a vertical CTE for the copper film of 22.8 ppm/K, somewhat higher than the measured 19.3 ppm/K. Since the measured value falls between this fully constrained calculation and the expected bulk value, we conclude that the films are only partially constrained by the substrate. This is unsurprising because of the relatively small deposition area (1.98 cm^2^), allowing the deposited film some freedom to expand laterally in response to the rising temperature.

### 3.4. Electrical Properties

**Figure 12:**
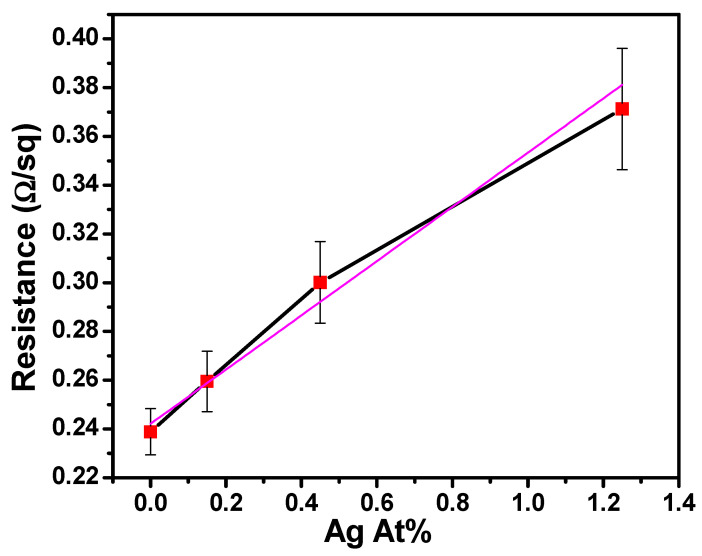
Sheet resistance vs. the Ag content of Cu(Ag) films showing that with an increase in the Ag content of the Cu(Ag) films, the sheet resistance also increases linearly. Black line connects means of data, with linear fit plotted in purple.

The sheet resistance values of the Cu(Ag) alloy films, plotted against the Ag content, are shown in Figure 12. In the case of Ag-free Cu, the measured sheet resistance stands at 0.239 Ω/sq, nearly double the sheet resistance reported in the literature for Cu films, approximately 0.108 Ω/sq [27]. The two times increase in resistance observed in our Cu film even in the absence of Ag can be attributed to the distinct chemistry and the presence of C and O impurities within the film. Furthermore, the surface roughness of the films contributes to additional electron scattering, which increases the likelihood of interactions between electrons and lattice vibrations (phonons), resulting in higher resistance.

Figure 12 also shows a linear increase in resistance with Ag content. The highest Ag content in the films, 1.25 at%, corresponds to the highest resistance value of 0.371 Ω/sq. The increase in resistance can be attributed to several factors in addition to the ones cited above, including impurity scattering at dissimilar atoms in the lattice. The introduction of Ag atoms can also cause changes in the film’s microstructure, such as the formation of grain boundaries or lattice defects, which impede electron flow and contribute to increased resistance. While thickness is typically a significant factor that can drastically affect the measured values, in our case, all deposited films were within the range of 220 to 250 nm, and we anticipate minimal differences in resistance due to this minor variation. Although substrate effects may exist, they should remain consistent across all samples due to their similar thickness. Taken together, these variations can account for the overall increase in resistance as the Ag content in the Cu(Ag) alloy films increases.

## 4. Conclusions

Cu-Ag alloys were deposited by the pulsed electrodeposition method using a sulfuric acid electrolyte. The alloy composition was determined by using SIMS and showed that Ag content depends on the duty cycle in the pulsed deposition. STEM-EDS revealed a uniform distribution of Ag throughout the thickness of the deposited film. The mechanical and physical properties of the films, including the hardness, modulus, CTE, and resistance, were also investigated and found to be linearly related to the Ag content. Our results demonstrate that Cu(Ag) alloy films have superior mechanical and physical properties for hybrid bonding compared to Ag-free Cu, with increasing Ag content leading to higher modulus, hardness, and CTE values, although these advantages come at the expense of increased electrical resistance. The tunability of these properties provides a strategic avenue for tailoring material properties in hybrid bonding scenarios, especially when superior mechanical resilience and increased CTE are sought. As we move forward, further experiments and measurements would be needed to optimize and integrate these alloys in the 300 mm fab, with this study (and forthcoming additional publications) serving to guide the design and optimization of Cu(Ag) alloy films for use in hybrid bonding applications.

## Figures and Tables

**Table 1:** Sample ID, deposition conditions, and respective Ag composition.

Sample ID	Duty Cycle (%)	Ag Content (At %)
Ag-free Cu	-	0
Cu(Ag)1	20	0.15
Cu(Ag)2	10	0.45
Cu(Ag)3	4	1.25

**Table 2:** Hardness and modulus values corresponding to the Ag composition of the alloy films.

Sample ID	Ag Content (At %)	Modulus (GPa)	Hardness (GPa)
Ag-free Cu	0	111	1.70
Cu(Ag)1	0.15	113	1.81
Cu(Ag)2	0.45	120	2.35
Cu(Ag)3	1.25	161	3.99

**Table 3:** CTE values corresponding to the Ag composition in the alloy films.

Sample ID	Ag Content (At %)	CTE (ppm/K)
Ag-free Cu	0	17.9
Cu(Ag)1	0.15	18
Cu(Ag)2	0.45	18.2
Cu(Ag)3	1.25	19.3

## Data Availability

Data are contained within the article.
